# Granzyme B Expression in the Tumor Microenvironment as a Prognostic Biomarker for Patients with Triple-Negative Breast Cancer

**DOI:** 10.3390/cancers15184456

**Published:** 2023-09-07

**Authors:** Kimihisa Mizoguchi, Hitomi Kawaji, Masaya Kai, Takafumi Morisaki, Saori Hayashi, Yuka Takao, Mai Yamada, Akiko Shimazaki, Tomofumi Osako, Nobuyuki Arima, Masayuki Okido, Yoshinao Oda, Masafumi Nakamura, Makoto Kubo

**Affiliations:** 1 Department of Surgery and Oncology, Graduate School of Medical Sciences, Kyushu University, 3-1-1 Maidashi, Higashi-ku, Fukuoka 812-8582, Japan; km10211089@yahoo.co.jp (K.M.); kawaji@med.kyushu-u.ac.jp (H.K.); mkbloom777@gmail.com (M.K.); smiley199903@yahoo.co.jp (T.M.); shayashisurg1@gmail.com (S.H.); takao.yuka.414@s.kyushu-u.ac.jp (Y.T.); kaneshiroyamada.mai@gmail.com (M.Y.);; 2 Breast Center, Kumamoto Shinto General Hospital, 3-2-65 Oe, Chuo-ku, Kumamoto 862-8655, Japan; 3 Department of Pathology, Kumamoto Shinto General Hospital, 3-2-65 Oe, Chuo-ku, Kumamoto 862-8655, Japan; 4 Department of Surgery, Hamanomachi Hospital, 3-3-1 Nagahama, Chuo-ku, Fukuoka 810-8539, Japan; 5 Department of Anatomic Pathology, Graduate School of Medical Sciences, Kyushu University, 3-1-1 Maidashi, Higashi-ku, Fukuoka 812-8582, Japan; oda.yoshinao.389@m.kyushu-u.ac.jp

**Keywords:** granzyme B, biomarker, triple-negative breast cancer, immune checkpoint inhibitors, programmed cell death ligand 1

## Abstract

**Simple Summary:**

Triple-negative breast cancer (TNBC) is generally malignant and has a poor prognosis. New biomarkers and therapeutic strategies are therefore needed. In this study, we investigated whether granzyme B (GZMB) in the tumor microenvironment can be a biomarker of therapeutic response and prognosis in TNBC via the immunohistochemical staining of clinical specimens from 230 patients with primary TNBC. The key results were in the programmed cell death ligand 1 (PD-L1)-positive group, where GZMB-high TNBC patients had better recurrence-free survival and overall survival than GZMB-low patients. A multivariate analysis also showed significantly better overall survival in PD-L1-positive and GZMB-high patients. These results indicate that GZMB is a useful prognostic marker in PD-L1-positive TNBC patients.

**Abstract:**

Tumor-infiltrating lymphocytes in the tumor microenvironment are important in the treatment of triple-negative breast cancer (TNBC). Cytotoxic T cells produce cytokines and cytotoxic factors, such as perforin and granzyme, which induce apoptosis by damaging target cells. To identify biomarkers of these cells, we investigated granzyme B (GZMB) in the tumor microenvironment as a biomarker of treatment response and prognosis in 230 patients with primary TNBC who underwent surgery without preoperative chemotherapy between January 2004 and December 2014. Programmed cell death ligand 1 (PD-L1) positivity was defined as a composite positive score ≥10 based on the PD-L1 immunostaining of tumor cells and immune cells. GZMB-high was defined as positivity in ≥1% of tumor-infiltrating lymphocytes (TILs). Among the 230 TNBC patients, 117 (50.9%) had CD8-positive infiltrating tumors. In the PD-L1-positive group, a Kaplan–Meier analysis showed that GZMB-high TNBC patients had better recurrence-free survival (RFS) and overall survival (OS) than GZMB-low patients and that OS was significantly longer (RFS: *p* = 0.0220, OS: *p* = 0.0254). A multivariate analysis also showed significantly better OS in PD-L1- and GZMB-high patients (hazard ratio: 0.25 (95% IC: 0.07–0.88), *p* = 0.03). Our findings indicate that GZMB is a useful prognostic biomarker in PD-L1-positive TNBC patients.

## 1. Introduction

Approximately fifteen percent of all breast cancer cases are triple-negative [1]. Triple-negative breast cancer (TNBC) is defined as not expressing estrogen receptors (ERs), progesterone receptors (PRs), or human epidermal growth factor receptor 2 (HER2). TNBC is treated with endocrine therapy and anti-HER2 therapy and chemotherapy. TNBC has a higher grade of malignancy, a higher frequency of distant metastases, and a poorer prognosis than other breast cancer subtypes [1]. Therefore, new treatment strategies and biomarkers are needed to improve treatment efficacy and the prognosis of TNBC patients.

The tumor microenvironment is composed of tumor tissue, surrounding normal tissue, and immune cells, and it plays a major role in tumor progression [2]. Studies on the tumor microenvironment indicate that tumor-infiltrating lymphocytes (TILs) play an important role in the treatment of cancers with a high tumor mutation burden, such as TNBC [3]. In addition, a relationship between TILs and the expression of programmed death-ligand 1 (PD-L1) has been demonstrated; a high TIL/PD-L1-positive population in TNBC is associated with a favorable prognosis [4], but the underlying mechanism of this association is unclear. 

**Figure 1:**
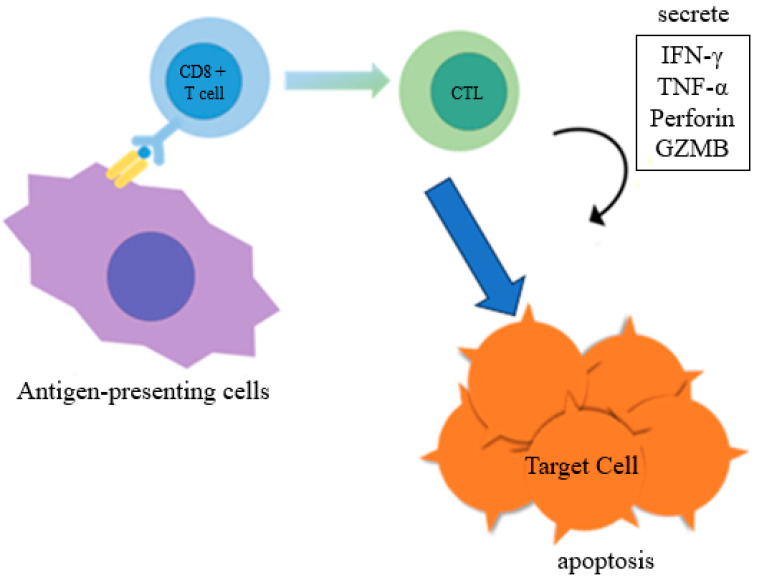
Cytotoxic T cells (CTLs) are regulated by the transcription factor T-bet, and they cause apoptosis by damaging target cells with cytokines and cytotoxic factors, such as perforin and granzyme B (GZMB). IFN-γ: interferon-gamma; TNF-α: tumor necrosis factor-alpha.

Cytotoxic T cells are activated by the transcription factor T-bet, and they release cytokines and cytotoxic agents, such as perforin A and granzyme B (GZMB), that damage target cells and cause apoptosis (Figure 1) [5]. GZMB is associated with liver cancer progression [6] and is a potential prognostic marker of colorectal cancer [7]. Furthermore, serial changes in GZMB expression in non-small-cell lung cancer may serve as a biomarker for monitoring monotherapy with immune checkpoint inhibitors [8,9]. In breast cancer immunotherapy, the role of GZMB in the tumor microenvironment is also important [10]. Mononuclear cells and lymphocytes infiltrate the tumor microenvironment of TNBCs, and a combination of immune checkpoint inhibitors and chemotherapy can be effective in high-risk TNBC patients. However, such therapy does not suit all patients; some do not respond and relapse. Therefore, biomarkers are needed to help identify patients who need additional chemotherapy or immunotherapy. In this study, we examined whether GZMB in the tumor microenvironment can be a biomarker for treatment response and prognosis in TNBC.

## 2. Materials and Methods

### 2.1. Patients and Tumor Samples

Patients with a diagnosis of primary TNBC who attended Kyushu University Hospital (Fukuoka, Japan), Hamanomachi Hospital (Fukuoka, Japan), and Kumamoto Municipal Hospital (Kumamoto, Japan) from January 2004 to December 2014 were selected. Clinical stage IV patients and patients who received neoadjuvant chemotherapy were excluded. Paraffin-embedded surgical biopsy material and clinical data from 230 patients were obtained. The patients received adjuvant treatment according to the National Comprehensive Cancer Network Guidelines for the treatment of breast cancer (http://www.nccn.org/professionals/physician_gls/f_guidelines.asp#breast (accessed on 1 November 2022)), the Japanese Breast Cancer Society Clinical Practice Guidelines for the systemic treatment of breast cancer (http://jbcs.xsrv.jp/guidline/ (accessed on 1 November 2022), in Japanese), and the recommendations of the St. Gallen International Breast Cancer Conference [11,12,13,14]. 

Informed consent was obtained from all participants. The study complied with the principles of the Declaration of Helsinki and was approved by the Institutional Review Board of Kyushu University Hospital (No. 30-231).

### 2.2. Pathological Assessment

All surgical specimens were fixed in a 10% neutral buffered formaldehyde solution for 6–72 h, embedded in paraffin, and sectioned at a thickness of 4 μm. Tumor subtypes were diagnosed via immunohistochemistry. ER-positive or PgR-positive tumors were defined as ≥1% positive staining for ER or PgR. HER2-positive tumors were defined as HER2 immunohistochemistry staining of 3+ according to standard criteria [15,16] or via fluorescence in situ hybridization when HER2 gene amplification was detected.

TILs were assessed using hematoxylin-and-eosin-stained sections according to the guidelines published by the International TILs Working Group [17,18]. Cases were defined as TILs-high if there were more than 50% stromal TILs, which is also known as lymphocyte-predominant breast cancer, and TILs-low if there were less than 50%.

### 2.3. Immunohistochemistry

A primary GZMB antibody (monoclonal mouse, GrB-7; Dako Inc., Glostrup, Denmark) was used at a 1:50 dilution as previously described [19]. Briefly, slides were deparaffinized, and antigens were retrieved using a solution (pH 6.0) at just below boiling point (95–98 °C). Sections were incubated with the GZMB primary antibody at optimized concentrations overnight at 4 °C and then with a secondary anti-mouse antibody for 40 min at room temperature. Immunostaining was visualized using 3,3′-diaminobenzidin (DAB), and then sections were counterstained with hematoxylin for 10 s. GZMB staining was evaluated in areas of TILs. The number of GZMB-positive cells was counted in a microscopic field at ×200 magnification (0.00625 mm^2^) in three randomly selected areas. The ratio of the average count of GZMB-positive cells to total TILs was calculated. The results were interpreted as positive when ≥1%. Ki-67 was dichotomized into Ki-67-high (Ki-67 labeling index > 20%) and Ki-67-low (Ki67 labeling index ≥ 20).

**Figure 2:**
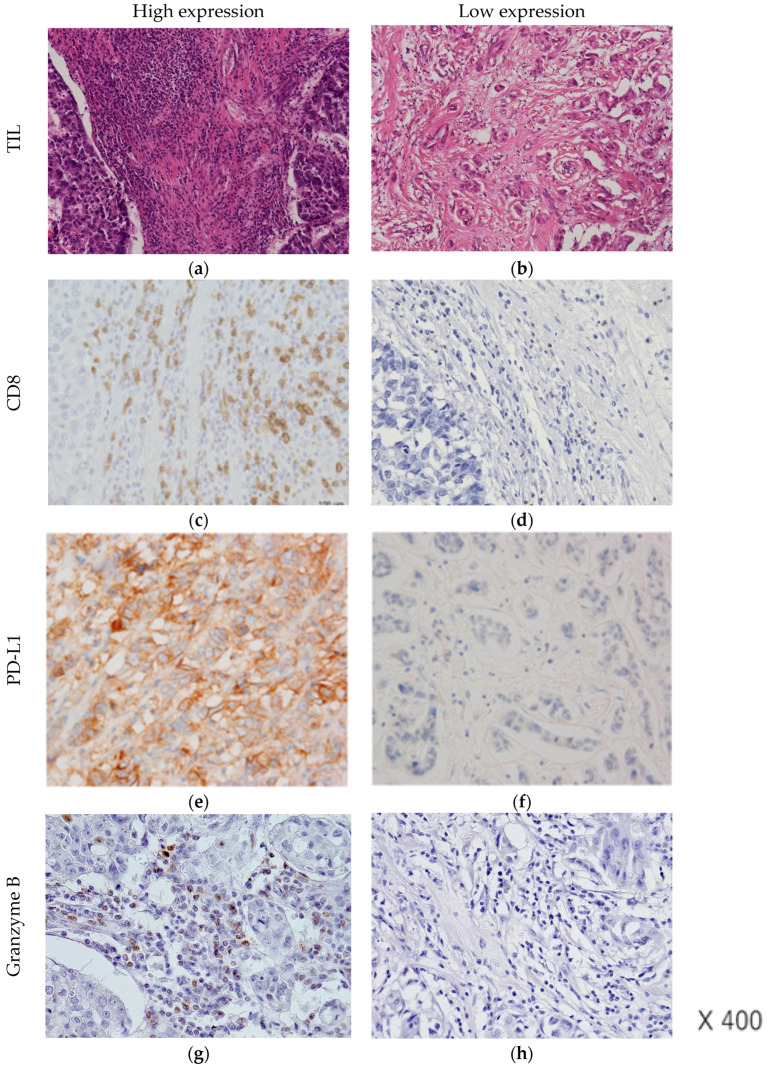
Immunohistochemistry for TIL, CD8, PD-L1, and GZMB in triple-negative breast cancer samples (magnification: ×400). (**a**): TIL-high, (**b**): TIL-low, (**c**): CD8-high, (**d**): CD8-low, (**e**): PD-L1-positive, (**f**): PD-L1-negative, (**g**): GZMB-high, (**h**): GZMB-low. TIL: tumor-infiltrating lymphocyte; CD8: cluster of differentiation 8; PD-L1; programmed cell death-ligand 1.

CD8-positive T cells were counted separately according to their intratumoral or stromal localization within a microscope field at 200× magnification (0.00625 mm^2^). Five areas with the most abundant infiltration were selected, and the average count was calculated. The results were interpreted as positive when there were more than or equal to 30 cells per 0.0625 mm^2^ in an intratumoral or stromal area [20]. A primary anti-PD-L1 antibody (monoclonal rabbit, E1L3N; Cell Signaling Technology, Beverly, MA, USA) was used at a 1:200 dilution. PD-L1 expression was evaluated using a combined positive score (CPS). The results were interpreted as positive when the CPS was ≥20 (Figure 2). In addition, PD-L1 immune cell positivity was defined as expression in ≥5% of tumor-infiltrating immune cells [12]. For T-bet, following the examination of multiple cutpoints, an absolute count of 30 positive intratumoral lymphoid cells (within or within close proximity of the epithelial cell nests) was used as the cutoff for positivity (T-bet/high). Tumors with lower levels or no T-bet intratumoral lymphoid cells were considered as T-bet/low [4,21].

### 2.4. Statistics

We used logistic regression to compare continuous variables. We also used the χ^2^ test to compare the categorical variables of GZMB-positive and -negative groups. The survival endpoints evaluated were recurrence-free survival (RFS) and overall survival (OS). RFS was defined as the time from surgery to recurrence, including both local relapse and metastatic disease. OS was defined as the time from surgery to the date of death from any cause. For RFS and OS, the Kaplan–Meier method was applied, and survival curves were compared using the log-rank test; values of *p* < 0.05 were considered statistically significant. A statistical analysis was performed using JMP 15 (SAS Institute Inc., Cary, NC, USA).

## 3. Results

**Figure 3:**
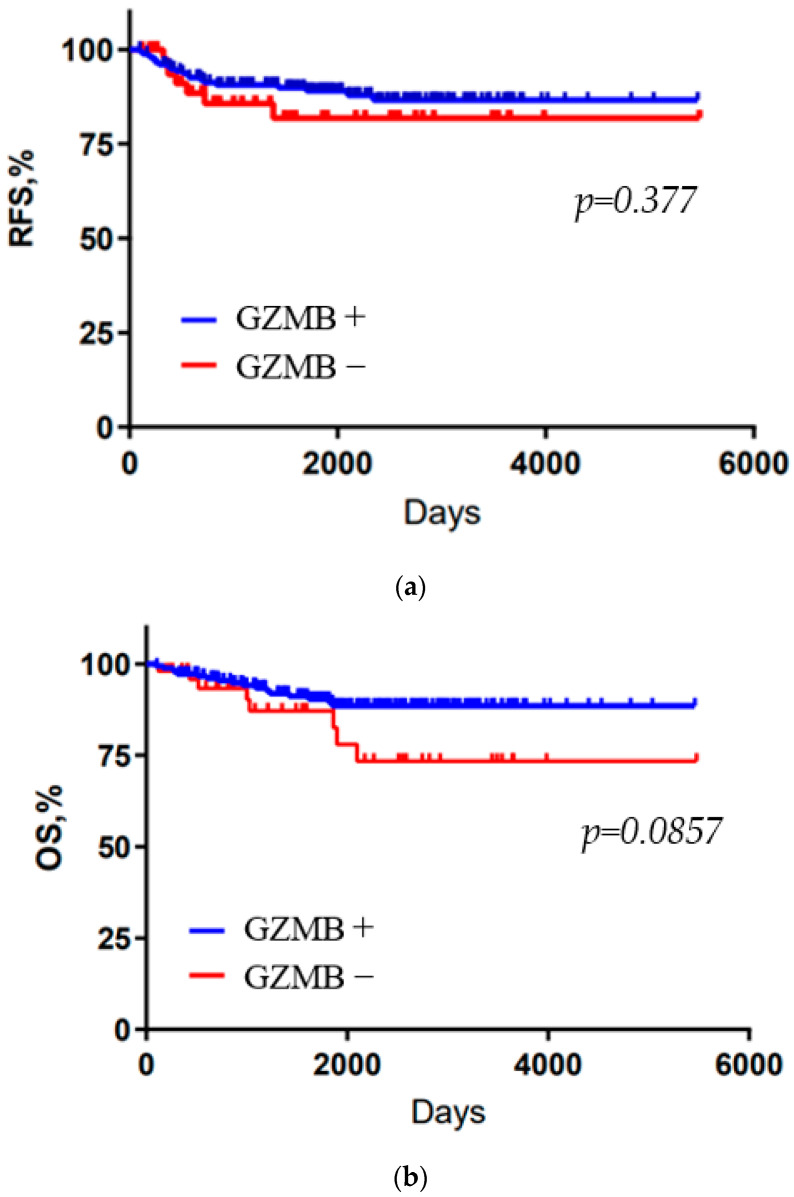
Kaplan–Meier analysis of recurrence-free survival (RFS) and overall survival (OS) in all TNBC patients. (**a**): RFS, (**b**): OS.

**Figure 4:**
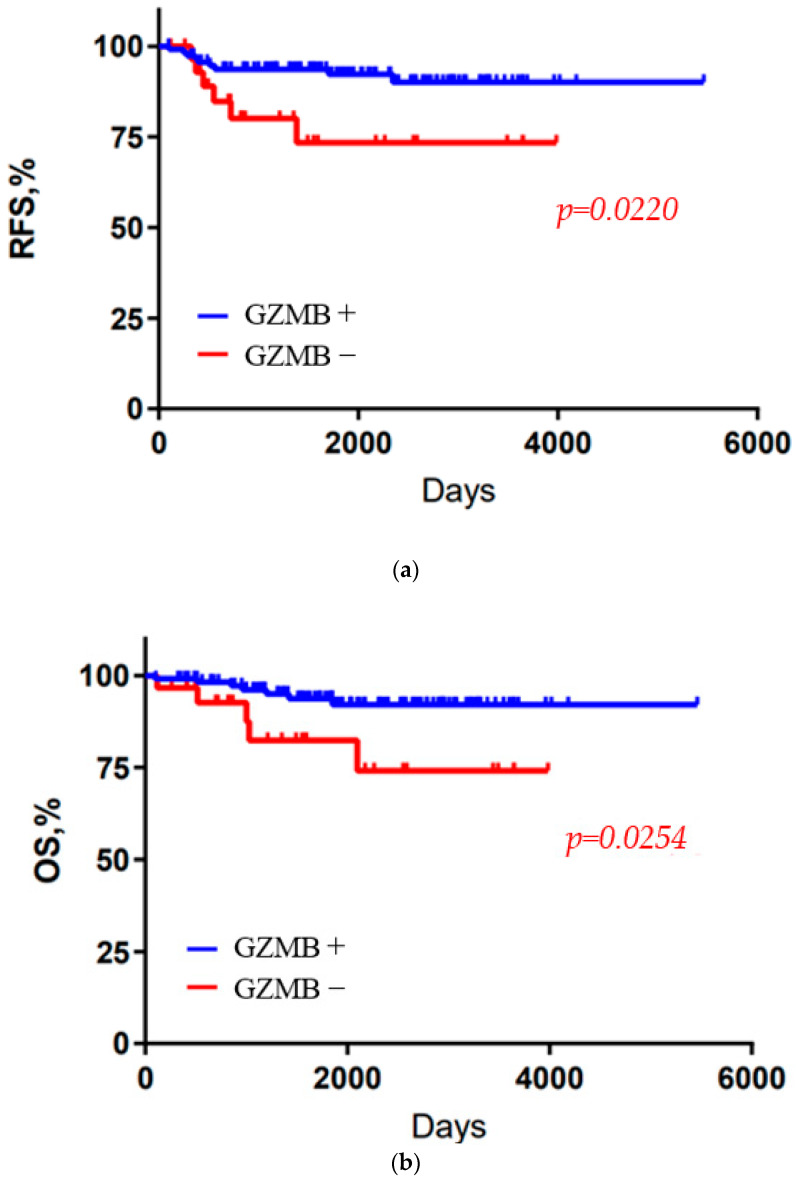
Kaplan–Meier analysis of GZMB recurrence-free survival (RFS) and overall survival (OS) in PD-L1-positive patients. (**a**): RFS, (**b**): OS.

Table 1 summarizes the age at diagnosis, tumor diameter, lymph node status, pathological stage, histological features, presence of TILs and CD8-positive T cells, and PD-L1 expression for the 230 TNBC patients (Table S1 summarizes patient characteristics by GZMB cut off value (1% or 5%)). The median age of all patients was 60 years. Tumor size was less than 2 cm, N factor was N0 in more than half of the patients, and pathological stage was stage I in about 70% of all patients. Among all TNBC patients, 159 (69.1%) had nuclear grade (NG) 3, and 178 (77.4%) had high Ki-67 (≥20%). Furthermore, 117 (50.9%) had TIL-high or CD8+ tumors, and 126 (54.8%) were PD-L1-positive. The immunohistochemical classification of 230 tumors revealed that 181 (78.7%) were GZMB-high and 49 (21.3%) were GZMB-low. There were no differences in tumor size, N factor, or pathological stage between the two groups. GZMB expression was significantly correlated with high TIL infiltration (*p* = 0.0006), CD8 (*p* = 0.002), and PD-L1 expression (*p* = 0.012) (Table 2). The Kaplan–Meier curves for the GZMB-high and GZMB-low groups are shown in Figure 3 and Figure 4. RFS and OS were not significantly prolonged in the GZMB-high group compared with those in the GZMB-low group (Figure 3). However, in the PD-L1-positive group, RFS and OS were significantly prolonged in the GZMB-high group compared with those in the GZMB-low group (RFS: *p* = 0.0220, OS: *p* = 0.0254) (Figure 4). Table 3 and Table 4 show the OS and RFS results of the PD-L1-positive group obtained via univariate and multivariate analyses (Table S2 summarizes the analysis by GZMB cut off value (1% or 5%). In the univariate analysis, GZMB, tumor diameter, and lymph nodes were associated with OS and RFS. In the multivariate analysis, GZMB was independently associated with RFS and OS (RFS: hazard ratio (HR) = 0.21, 95% confidence interval (CI) 0.07–0.69, *p* = 0.009; OS: HR = 0.25, 95% CI 0.69, *p* = 0.009; HR CI 0.07–0.88, *p* = 0.03).

## 4. Discussion

The purpose of this study was to investigate whether GZMB can be a biomarker for therapeutic efficacy and prognosis in TNBC. We demonstrated that GZMB expression significantly correlated with high TIL infiltration, CD8 expression, and PD-L1 expression in a relatively large cohort of TNBC patients. Furthermore, in the PD-L1-positive group, high-GZMB-expressing TNBCs had significantly longer RFS and OS than low-GZMB-expressing TNBCs. Significant results were also demonstrated by a multivariate analysis (Table 3 and Table 4).

GZMB can serve as a biomarker to predict responses to immune checkpoint inhibitors (ICIs) [22,23,24]. ICIs inhibit T effector cell replacement by T regulatory cells, activate dendritic cells, and activate tumor-infiltrating cytotoxic CD8+ and CD4+ T cells [20]. Several studies indicate that GZMB-specific positron emission tomography can be used to determine therapeutic responses to chemotherapy and ICI in vivo [10,22,25]. To further explore the usefulness of GZMB as a biomarker, we assessed tissues from 230 TNBC patients. We evaluated TILs via HE staining and the immunohistochemical localization of CD8, PD-L1, and GZMB.

**Figure 5:**
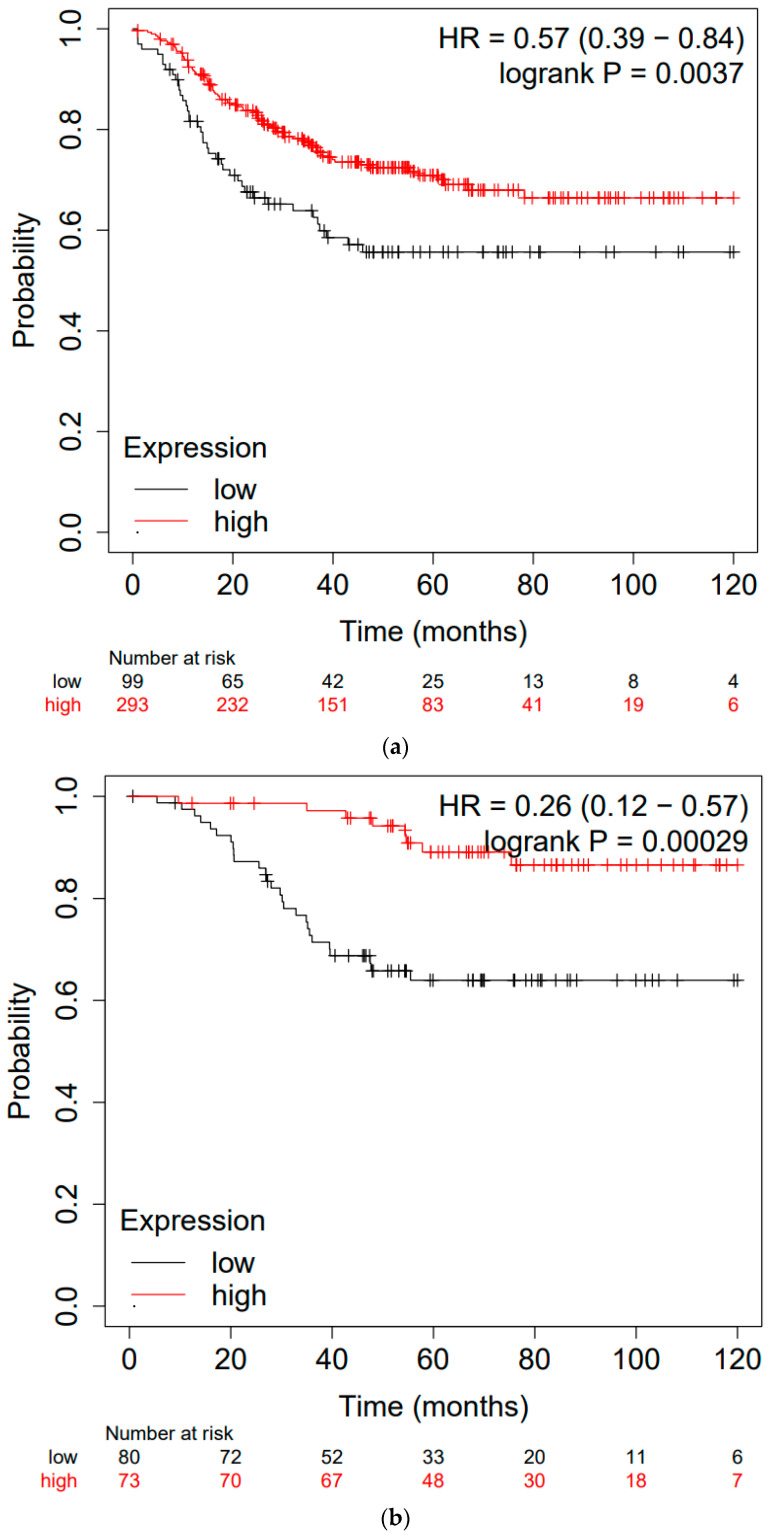
Kaplan–Meier analysis of recurrence-free survival (RFS) and overall survival (OS) in all TNBC patients in KM-plotted data. (**a**): RFS, (**b**): OS.

It is well known that the tumor microenvironment plays an important role in immunochemotherapy. TNBCs are particularly immunogenic, having high numbers of TILs and high levels of PD-L1 expression [26,27]. As expected, in the present study, high rates of CD8-positive TILs and high levels of PD-L1 expression in tumors and immune cells were significantly correlated with GZMB expression. Our data showed no significant difference in OS and RFS between the high-GZMB-expression and low-GZMB-expression groups in the overall patient population, but significant differences were observed in PD-L1-positive patients; the KM-plotted data (Figure 5) showed significant differences in the overall patient population. These results indicate that the combination of chemotherapy and ICI may be more effective for patients with TNBC.

The pretreatment of T-cell infiltration, high PD-L1 expression, microsatellite instability, and high tumor mutational burden are thought to enhance the immunochemotherapy response [28,29,30]. Extracellular GZMBs generally have a biological half-life of 14 days, making them stable targets for immune activation [10]. Our results indicate that GZMB may serve as a biomarker for predicting treatment response and prognosis for advanced breast cancer.

This study has several limitations. First, the data did not include each patient’s treatment. In particular, it was not verified whether all patients in the PD-L1-positive group received chemotherapy and ICI. It is difficult to collect pre- and post-treatment tissues for immunohistochemical staining. Our ultimate goal is to evaluate GZMB as a specific biomarker of TNBC that can predict the response to ICI treatment. For this purpose, non-invasive biopsies, such as liquid biopsies, should be considered in the future. Second, the cohort was collected retrospectively. Prospective studies are inherently more accurate.

## 5. Conclusions

Our study showed that GZMB can be a prognostic predictor in PD-L1-positive TNBC patients. Therefore, the expression of GZMB in combination with PD-L1 may be an essential biomarker of the tumor immune system. Further studies are needed to investigate the potential of GZMB as a predictor of ICI and chemotherapy efficacy for PD-L1-positive TNBCs.

## Figures and Tables

**Table 1:** Clinicopathologic characteristics of patients with TNBC.

	Number of Patients	
	n = 230	
Age at diagnosis (y), mean (range)	60	(30–89)
Tumor size		
T1	131	57.0%
T2	90	39.1%
T3	8	3.5%
T4	1	4.3%
Nodal status		
N0	152	66.1%
N1	61	26.5%
N2	10	4.3%
N3	7	3.0%
Pathological stage		
Ⅰ	152	66.1%
Ⅱ	76	33.0%
Ⅲ	2	8.7%
Nuclear grade		
1/2	65	28.3%
3	159	69.1%
Unknown	6	2.6%
Ki67		
≤20	27	11.7%
>20	178	77.4%
Unknown	25	10.9%
TIL		
High	117	50.9%
Low	112	48.7%
CD8 + T cell		
High	117	50.9%
Low	113	49.1%
PD-L1 (CPS10)		
+	126	54.8%
−	104	45.2%

**Table 2:** Clinicopathological characteristics of TNBC patients divided into high- and low-GZMB-expression groups.

	Granzyme B-High		Granzyme B-Low		
	(n = 181)		(n = 49)		*p*-Value
Tumor size					
T1	99	54.7%	32	65.3%	0.5579
T2	74	40.9%	16	32.7%	
T3	7	3.9%	1	2.0%	
T4	1	0.6%	0	0.0%	
Nodal status					
N0	117	64.6%	35	71.4%	0.651
N1	50	27.6%	11	22.4%	
N2	9	5.0%	1	2.0%	
N3	5	2.8%	2	4.1%	
Pathological stage					
Ⅰ	117	64.6%	35	71.4%	0.3956
Ⅱ	63	34.8%	13	26.5%	
Ⅲ	1	0.6%	1	2.0%	
Nuclear grade					
1/2	48	26.5%	17	34.7%	0.2242
3	129	71.3%	30	61.2%	
Unknown	4	2.2%	2	4.1%	
Ki67					
≤20	21	11.6%	6	12.2%	0.6496
>20	145	80.1%	33	67.3%	
Unknown	15	8.3%	10	20.4%	
TIL					
High	81	44.8%	36	73.5%	**0.0006**
Low	99	54.7%	13	26.5%	
CD8 + T cell					
High	102	56.4%	15	30.6%	**0.002**
Low	79	43.6%	34	69.4%	
PD-L1(CPS10)					
+	104	57.5%	22	44.9%	**0.01171**
−	77	42.5%	27	55.1%	

**Table 3:** Factors associated with overall survival in the PD-L1-positive groups.

OS				
	Univariate		Multivariate	
	HR (95% CI)	*p* Value	HR (95% CI)	*p* Value
GZMB (high)	0.28 (0.09–0.89)	**0.04**	0.25 (0.07–0.88)	**0.03**
CD8 (positive)	1.76 (0.47–6.49)	0.38		
TIL (high)	0.47 (0.15–1.46)	0.19		
Tumor size (>2 cm)	3.26 (0.98–10.84)	**0.04**	3.70 (1.02–13.36)	0.05
Lymph node (positive)	4.06 (1.22–13.51)	**0.02**	3.19 (0.92–11.02)	0.06

**Table 4:** Factors associated with relapse-free survival in the PD-L1-positive groups.

	Univariate		Multivariate	
	HR (95% CI)	*p* Value	HR (95% CI)	*p* Value
GZMB (high)	0.27 (0.09–0.72)	**0.009**	0.21 (0.07–0.69)	**0.009**
CD8 (positive)	1.88 (0.60–5.83)	0.25		
TIL (high)	0.47 (0.15–1.46)	0.19		
Tumor size (>2 cm)	2.87 (1.04–7.90)	**0.04**	3.08 (1.06–8.92)	**0.04**
Lymph node (positive)	3.32 (1.20–9.17)	**0.02**	2.53 (0.86–7.42)	0.09

## Data Availability

The authors select to not share data.

## Supplementary Materials

The following supporting information can be downloaded at https://www.mdpi.com/article/10.3390/cancers15184456/s1, Table S1: Patients’ characteristics; Table S2: Univariate analysis.
